# Delayed breastfeeding initiation and infant survival: A systematic review and meta-analysis

**DOI:** 10.1371/journal.pone.0180722

**Published:** 2017-07-26

**Authors:** Emily R. Smith, Lisa Hurt, Ranadip Chowdhury, Bireshwar Sinha, Wafaie Fawzi, Karen M. Edmond, on behalf of the Neovita Study Group

**Affiliations:** 1 Department of Global Health and Population, Harvard T. H. Chan School of Public Health, Boston, MA, United States of America; 2 Division of Population Medicine, Cardiff University School of Medicine, Wales, United Kingdom; 3 Centre for Health Research and Development, Society for Applied Studies, New Delhi, India; 4 Department of Nutrition, Harvard T. H. Chan School of Public Health, Boston, MA, United States of America; 5 Department of Epidemiology, Harvard T. H. Chan School of Public Health, Boston, MA, United States of America; 6 School of Paediatrics and Child Health, University of Western Australia, Perth, Australia; Centre Hospitalier Universitaire Vaudois, FRANCE

## Abstract

**Objective:**

To assess the existing evidence regarding breastfeeding initiation time and infant morbidity and mortality.

**Study design:**

We conducted a systematic review and meta-analysis. We searched Pubmed, Embase, Web of Science, CINAHL, Popline, LILACS, AIM, and Index Medicus to identify existing evidence. We included observational studies and randomized control trials that examined the association between breastfeeding initiation time and mortality, morbidity, or nutrition outcomes from birth through 12 months of age in a population of infants who all initiated breastfeeding. Two reviewers independently extracted data from eligible studies using a standardized form. We pooled effect estimates using fixed-effects meta-analysis.

**Results:**

We pooled five studies, including 136,047 infants, which examined the association between very early breastfeeding initiation and neonatal mortality. Compared to infants who initiated breastfeeding ≤1 hour after birth, infants who initiated breastfeeding 2–23 hours after birth had a 33% greater risk of neonatal mortality (95% CI: 13–56%, I^2^ = 0%), and infants who initiated breastfeeding ≥24 hours after birth had a 2.19-fold greater risk of neonatal mortality (95% CI: 1.73–2.77, I^2^ = 33%). Among the subgroup of infants exclusively breastfed in the neonatal period, those who initiated breastfeeding ≥24 hours after birth had an 85% greater risk of neonatal mortality compared to infants who initiated <24 hours after birth (95% CI: 29–167%, I^2^ = 33%).

**Conclusions:**

Policy frameworks and models to estimate newborn and infant survival, as well as health facility policies, should consider the potential independent effect of early breastfeeding initiation.

## Introduction

Five million deaths in children younger than five years were reported globally in 2015; almost half (46%) of these occurred in the neonatal period [1]. An even a greater number of children are affected by prematurity, malnutrition, and septicaemia, which can result in serious physical and neurological sequelae [2]. Interventions that can be implemented at scale, starting before birth and continuing throughout the postnatal period, are needed to reduce mortality and morbidity in children and young infants [2]. Currently, only 50% of infants in the world are breastfed during the first hour of life, and 60% are exclusively breastfed [3]. The World Health Organization (WHO) recommends that newborns initiate breastfeeding within one hour of birth, but this recommendation is not supported by an official WHO guideline. Additional evidence is needed to inform public health investment and to facilitate the implementation of breastfeeding promotion programs.

Systematic reviews published in 2013 and 2015 reported that early breastfeeding initiation (defined in these reviews as initiation within 24 hours of birth) was associated with reduced neonatal mortality [4, 5]. However, no association was found in a subgroup analysis which examined risks among exclusively breastfed infants [4], and early breastfeeding initiation was not included as an independent intervention in the recent Lancet 2013 Nutrition Series [6]. Substantial data on the association between early breastfeeding initiation and neonatal mortality has recently become available [7–11], including new data from large cohorts of mothers and infants who participated in three neonatal vitamin A trials in Ghana, India, and Tanzania [12–14]. Data from these cohorts was used to examine the association between very early breastfeeding initiation (defined as initiation within one hour of birth) and neonatal and post-neonatal mortality. These trials have also provided new data regarding early initiation among exclusively breastfed infants [15].

This paper reports the results of a systematic review of all studies published through December 2015 and updates pooled estimates of associations between delayed breastfeeding initiation and neonatal mortality. We assessed the relationship between very early initiation of breastfeeding (within one hour of birth) compared to delayed initiation (2–23 hours and 24 hours or more after birth) on neonatal mortality (<28 days). We also compared breastfeeding initiation within 24 hours of birth to initiation 24 hours or more after birth in order to update the results of the previous meta-analyses. We further examined the relationship between breastfeeding initiation time and infant morbidity and growth.

## Methods

### Protocol and registration

The protocol for this review was developed by the co-authors after examining existing review articles. We registered the protocol with the International Prospective Register of Systematic Reviews (PROSPERO) (Registration Number CRD42015032321). We followed MOOSE Guidelines for the meta-analysis of observational data while conducting the search, analysis and writing the manuscript [16].

### Inclusion and exclusion criteria

We included observational studies (*e*.*g*. cross-sectional studies, cohort studies, and case-control studies) and randomized control trials, if they examined the association between breastfeeding initiation time and mortality, morbidity, or nutrition outcomes from birth through 12 months of age in a population of infants who all initiated breastfeeding. There were no date restrictions. Studies were excluded if they were non-human studies, case reports or case study designs, or if the paper was published in abstract form only.

### Definitions

The exposure of interest was breastfeeding initiation time. We assessed the relationship between very early initiation (within one hour of birth) compared to delayed initiation (2–23 hours and 24 hours or more after birth). We also compared breastfeeding initiation less than 24 hours to 24 hours or more.

Specific mortality outcomes of interest included: neonatal mortality (<28 days), infant mortality through six months (<180 days), and infant mortality through 12 months (<360 days). Specific morbidity and nutrition outcomes of interest included: diarrhea, respiratory infection, sepsis, omphalitis, hypothermia, weight loss, weight-for-age (WAZ), length-for- age (LAZ), weight-for-length (WLZ), and hospitalization.

### Search

We conducted electronic searches from December 9–15, 2015. We searched Pubmed, Embase, Web of Science, CINAHL, Popline, LILACS, AIM, and Index Medicus for the Eastern Mediterranean Region. The search strategy included: (i) terms to identify papers regarding breastfeeding, AND (ii) terms to identify papers regarding timing OR initiation, AND (iii) terms for mortality OR morbidity outcomes. There were no date restrictions. The full search strategy used for each database is available in S1 Text.

### Study selection, data collection, and quality assessment

Two reviewers independently assessed the titles and abstracts of all studies, removed duplicates, and categorized each paper as eligible, ineligible, or unclear using the eligibility criteria defined above. Disagreements were resolved through consultation with a third reviewer. Two reviewers independently extracted data for all studies that met the inclusion criteria including: study characteristics, study quality, and the effect estimates showing the relationship between breastfeeding initiation time and infant morbidity and mortality. When available, we used mortality estimates that excluded deaths in the first two to four days of life in order to rule out reverse causality. The quality of included studies was assessed using criteria developed in accordance with the World Health Organization (WHO) Child Health Epidemiology Reference Group (CHERG) (Table 1) and the overall quality of evidence was assessed using GRADE guidelines [17, 18].

**Table 1:** Criteria used to classify the quality of included studies.

	Study Design	Selection Bias	Information Bias	Attrition bias	Confounding	Reverse Causality
**High**	RCT	Population-based recruitment	Assessed exposure within 30 days of birth and prior to outcome	Loss to follow up <10%	Model adjusts for gestational age or low birthweight. Other adjustments desirable.	Must exclude early infant deaths or those who unable to initiate breastfeeding early.
**Moderate**	-			Loss to follow up 10-<15%		
**Low**	Observational	Cross-sectional recruitment	Assessed exposure more than 30 days after birth or after outcome occurred	Loss to follow up 15-<20%		
**Very Low**	-			Loss to follow up >20%	-	-

### Analyses

We pooled relative risks and 95% confidence intervals for all outcomes with two or more included studies. Because no heterogeneity was apparent, data synthesis was conducted using fixed effects meta-analysis. Heterogeneity of effects were assessed visually using Forest Plots of relative risks, quantified by the I^2^, and tested by the Q statistic tests [19]. Q tests with p values <0.05 or I^2^ values >50% were considered to represent substantial heterogeneity. All analyses were done using STATA 14 software.

We planned to use stratified meta-analyses to explore potential sources of heterogeneity on the association between breastfeeding initiation and infant mortality and morbidity. We defined the following subgroups *a priori*: study quality (comparing high quality studies to low and medium quality studies); low birthweight (<2500 g) compared to normal birthweight infants; exclusively breastfed in the neonatal period compared to not exclusively breastfed infants (including partial and predominant breastfeeding); high income countries (HIC) compared to low- and middle-income countries (LMIC) (as defined by the World Bank; and HIV-exposed infants compared to HIV-unexposed infants.

## Results

**Fig 1:**
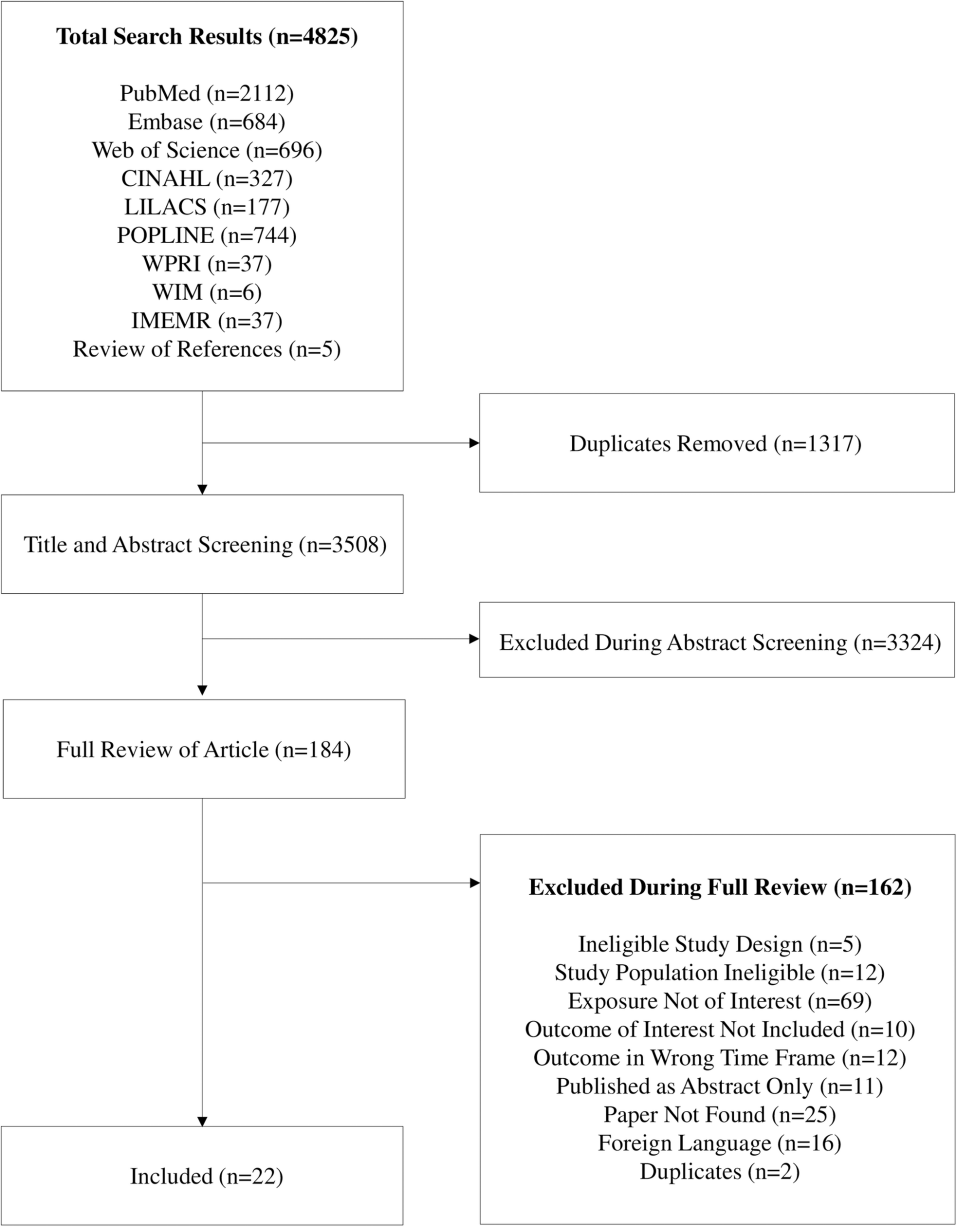
Flow diagram of search results and screening process.

We found a total of 4825 records. After removing 1317 duplicates, we screened 3508 titles and abstracts. 184 papers were selected for full text screening (Fig 1). A total of 22 papers were eligible for inclusion in the analysis [7–11, 15, 20–35] (S1 Table). Three papers referred to the same study and study population [25–27], and they were subsequently considered as one study, “Edmond 2006”. One paper contained pooled data from three studies [15], and we requested study-specific estimates from the authors so that each site could be included individually [12–14]. One study was categorised as high quality [35], seven studies were considered of moderate quality [9, 12–15, 27, 30–33], and 12 studies were considered low or very low quality [7, 8, 10, 11, 20–24, 28, 29, 34] (S2 Table).

**Fig 2:**
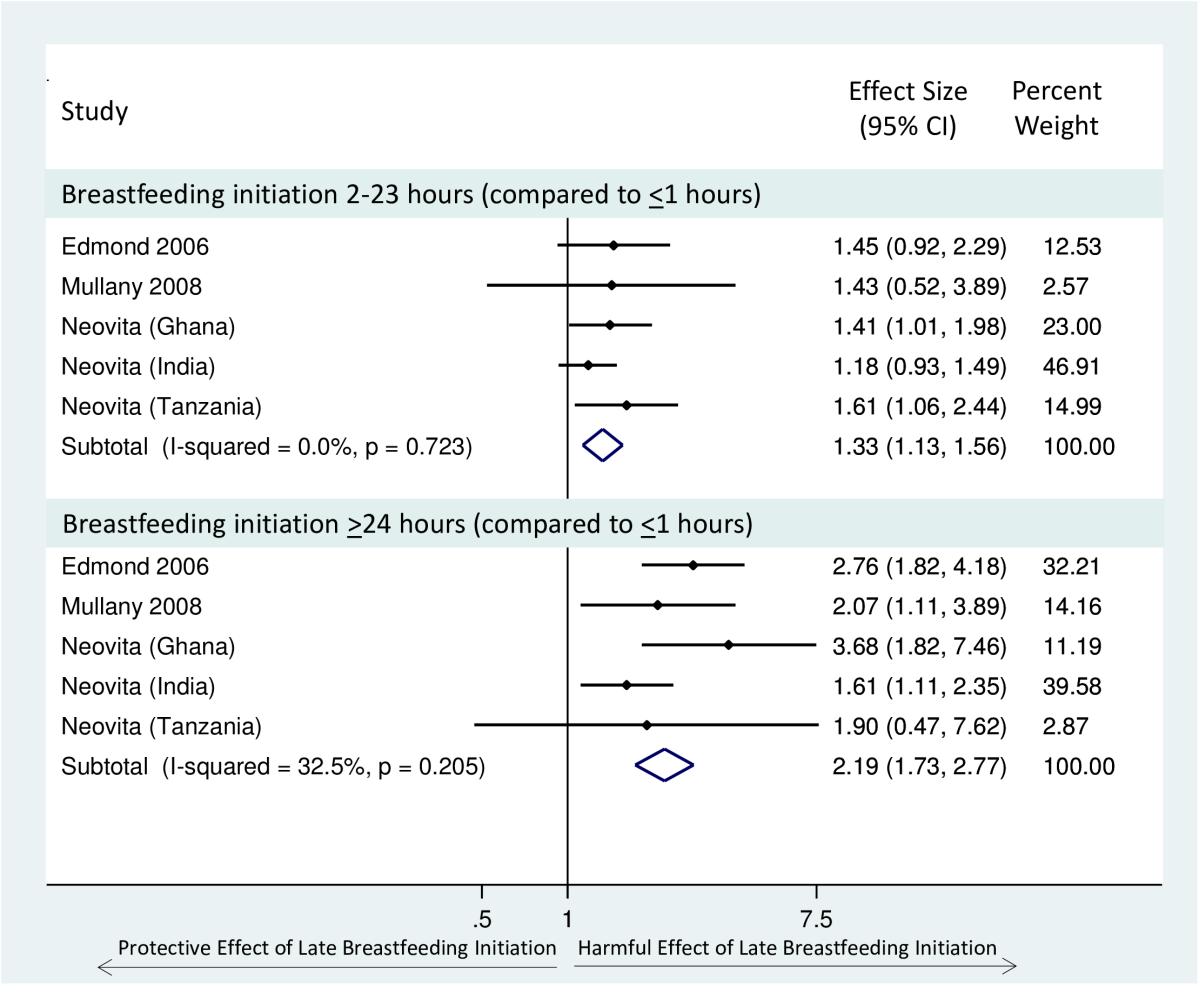
Forest Plot of the relative risk of neonatal mortality (excluding deaths in the first 2–4 days) for infants who initiated breastfeeding 2–23 hours or >24 hours after birth, compared to those who initiated breastfeeding early (<1 hour).

We identified 11 studies which examined breastfeeding initiation time and neonatal mortality [9, 10, 12–15, 20, 21, 27, 30, 33, 34] (Table 2). We pooled five of these studies which examined the association between delayed breastfeeding initiation (2–23 hours or ≥24 hours) compared to very early initiation (≤1 hour) on neonatal mortality, including a total of 136,047 infants [12–15, 27, 33]. There was evidence of a dose response relationship; increasing delay in breastfeeding initiation time was associated with an increasing risk of neonatal mortality. Infants who initiated breastfeeding 2–23 hours after birth had a 33% greater risk of neonatal mortality (95% CI: 13–56%), and infants who initiated breastfeeding ≥24 hours after birth were more than twice as likely to die during the neonatal period (pooled RR 2.19, 95% CI: 1.73–2.77) when compared to those who initiated breastfeeding within one hour of birth. There was no evidence of heterogeneity of effect (Fig 2). All pooled studies were categorised as ‘moderate’ quality. In a sensitivity analysis, we included estimates from Garcia *et al*. 2011 [30], which defined ‘early initiation’ as breastfeeding initiation <12 hours, and we found similar results (S1 Fig).

**Table 2:** Summary of studies of the association between early breastfeeding initiation and neonatal mortality. (*Reference group).

Study	N	Study Design	Exposure Definition	Effect Estimate (95% CI)	Quality
Neovita (India)	44,984	Prospective Cohort	Early (<1 hr)* vs. Late (2–23, ≥24 hrs)	aRR(2–23 hrs): 1.18 (0.93–1.49)	Moderate
			breastfeeding initiation	aRR(>24 hrs): 1.61 (1.11–2.35)	
					
Neovita (Ghana)	22,955	Prospective Cohort	Early (<1 hr)* vs. Late (2–23, ≥24 hrs)	aRR(2–23 hrs): 1.41 (1.01–1.98)	Moderate
			breastfeeding initiation	aRR(>24 hrs): 3.68 (1.82–7.46)	
					
Neovita (Tanzania)	31,999	Prospective Cohort	Early (<1 hr)* vs. Late (2–23, ≥24 hrs)	aRR(2–23 hrs): 1.61 (1.06–2.44)	Moderate
			breastfeeding initiation	aRR(>24 hrs): 1.90 (0.47–7.62)	
					
Akter 2015	3,190	Cross-sectional	Early (<1 hr) vs. Late* (>1 hr)	aOR: 0.86 (0.41–1.82)	Very Low
			breastfeeding initiation		
					
Shah 2014	6,399	Prospective Cohort.	Early (<1 hr) vs. Late* (>1 hr)	aRR: 0.7 (: 0.6–1.0)	Moderate
		Preterm infants only.	breastfeeding initiation		
					
Sutan 2014	500	Case Control. Low	Early (<1 hr)* vs. Late (>1 hr)	aOR: 2.03 (: 1.09–3.90)	Very Low
		birthweight infants only.	breastfeeding initiation		
					
Niswade 2011	1087	Prospective Cohort. Tribal infants only.	Early* vs. Late	aOR (tribal): 3.1 (05% CI:0.9–10.1)	Very Low
			breastfeeding initiation		
					
Garcia 2011	10,352	Prospective Cohort	Early (<12 hr)* vs. Late (12–23, ≥24 hrs)	aRR(12–24 hrs): 0.93 (: 0.59–1.46)	Moderate
			breastfeeding initiation	aRR (>24 hrs): 1.76 (: 1.01–3.07)	
					
Edmond 2006	10,942	Prospective Cohort	Early (<1 hr)* vs. Late (2–23 hrs, Day 2,	aOR(Day1): 1.45 (0.90–2.35)	Moderate
			Day 3, >Day 4)	aOR(Day2): 2.70 (1.70–4.3)	
			breastfeeding initiation	aOR(Day3): 3.01 (1.70–5.38)	
				aOR(>Day4): 4.42 (1.76–11.09)	
					
Bamji 2008	4,357	Case Control	Early (Day 1) vs. Late (Day 2, ≥Day 3)	OR(Day2): 1.58 (0.17–14.51)	Very Low
			breastfeeding initiation	OR(>Day3): 10.14 (3.17–32.42)	
					
Mullany 2008	22,838	Prospective Cohort	Early (<1 hr)* vs. Late (2–23 hrs, Day 2,	aOR(Day1): 1.43 (0.52–3.89)	Moderate
			Day 3, >Day 4)	aOR(Day2): 1.78 (0.64–5.00)	
			breastfeeding initiation	aOR(Day3): 2.43 (0.86–6.90)	
				aOR(>Day4): 2.06 (0.62–6.82)	

**Fig 3:**
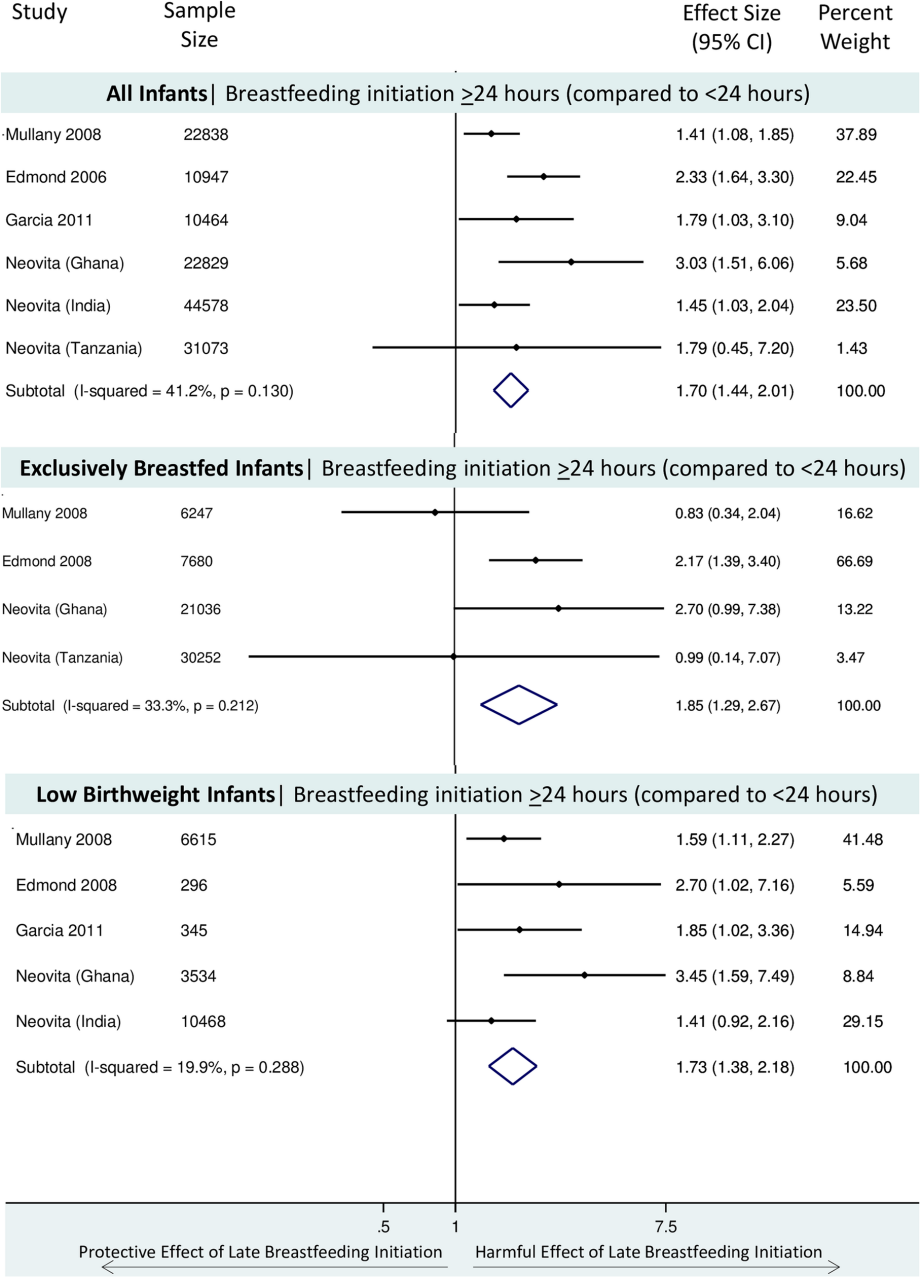
**Forest Plot of the relative risk of neonatal mortality (excluding deaths in the first 2–4 days) for infants who initiated breastfeeding >24 hours after birth, compared to those who initiated breastfeeding early (<24 hours) for i) all infants, ii) among exclusively breastfed infants, iii) among low birthweight infants**.

Six studies examined the association between breastfeeding initiation within 24 hours compared to ≥24 hours on neonatal mortality, including a total of 142,729 infants [12–15, 27, 30, 33]. In the largest cohorts relatively few infants initiated breastfeeding after 24 hours: 302 infants in Ghana, 236 infants in Tanzania, and 4,039 infants in India. Infants who initiated breastfeeding more than 24 hours after birth had a 70% greater risk of neonatal mortality compared to infants who initiated breastfeeding within 24 hours after birth (pooled RR 1.70, 95% CI: 1.44–2.01) (Fig 3). There was no evidence of substantial heterogeneity of effect (X^2^ p value = 0.13, I^2^ = 41%).

It was only possible to examine the association between very early initiation (<1 hour) and neonatal mortality among exclusively breastfed infants in three studies [12–14], and pooled estimates including these three cohorts have previously been published [15]. However, there were five studies which examined the relationship between breastfeeding initiation ≥24 hours compared to <24 hours and neonatal mortality among exclusively breastfed infants. The effect size was incalculable in the large India site [12], as there were no deaths among the small group of exclusively breastfed infants initiating breastfeeding ≥24 hours (n = 150). Thus, we pooled the estimates from four studies, including a total of 65,215 infants [13–15, 27, 33]. Infants who were exclusively breastfed in the neonatal period who delayed breastfeeding initiation 24 hours or more after birth had an 85% increased risk of neonatal mortality compared to infants who initiated breastfeeding early (<24 hours after birth) (pooled RR 1.85, 95% CI: 1.29–2.67) (Fig 3). There was no evidence of heterogeneity of effect (X^2^ p value = 0.21, I^2^ = 33%).

The same five studies included data which allowed examination of the relationship between delayed breastfeeding initiation (≥24 hours) compared to <24 hours and neonatal mortality among low birth weight infants. However, the effect was incalculable in the Tanzania site [14] as there were no deaths among the small group of low birthweight infants initiating breastfeeding after 24 hours (n = 35). Thus, we pooled the estimates from four studies, including a total of 21,258 infants [12, 13, 15, 27, 33]. Low birthweight infants who initiated breastfeeding more than 24 hours after birth had a 73% greater risk of neonatal mortality compared to infants who initiated breastfeeding <24 hours after birth (pooled RR 1.73, 95%CI: 1.38–2.18) (Fig 3). There was no evidence of heterogeneity of effect (X^2^ p value = 0.29, I^2^ = 20%).

We were unable to perform other subgroup analyses (*e*.*g*. by quality score; high-, middle-, or low-income status of country; maternal HIV status; etc.) as there were less than two studies in each subgroup strata. Only one study (including the three neonatal vitamin A trial cohorts) presented effect estimates for early infant mortality (one to three months and three to six months) [15], and no studies presented effect estimates for infant mortality through 12 months.

Table 3 summarizes the combined evidence regarding the association between delayed breastfeeding initiation and neonatal mortality. The overall quality of the evidence illustrating an increased risk of death among infants that initiate breastfeeding more than one hour after birth is rated as “high” quality. Although the pooled effect size is based on observational studies, the quality rating was upgraded because there is an apparent dose response relationship and a large increased risk of death (RR >2.0) for infants initiating ≥24 hours after birth. The other analyses that compared the risk of neonatal mortality for those initiating breastfeeding ≥24 hours after birth to those initiating <24 hours after birth among all infants, exclusively breastfed infants, and low birthweight infants, were classified as “moderate” in overall quality. The evidence is based on high-quality observational studies (which are considered to be “moderate” in quality due to the inherent limitations of observational studies), and the quality was not upgraded because a potential dose response relationship is not examined and the pooled relative risks are less than two.

**Table 3:** Summary of findings regarding the association between delayed breastfeeding association and neonatal mortality.

Outcome	Population	Illustrative comparative risks (95% CI)		Relative effect (95% CI)	Number of participants (studies)	Quality of the evidence (GRADE)^3^	
		Assumed risk^1^ Early Breastfeeding	Corresponding risk^2^ Delayed Breastfeeding				
Neonatal Mortality	All infants, who ever initiated breastfeeding, surviving 2–4 days		2–23 Hours:			High^4^	
			6.9 per 1000	(2–23 Hours):			
		<1 Hour:	(5.9 to 8.1)	1.33 (1.13–1.56)	136,047		
		5.2 per 1000			(5 studies)		
			>24 Hours:	(>24 Hours):			
			11.4 per 1000	2.19 (1.73–2.77)			
			(9.0 to 14.4)				
					
	All infants, who ever initiated breastfeeding, surviving 2–4 days	<24 Hours:	≥24 Hours:		142,729	Moderate^5^	
		7.7 per 1000	13.1 per 1000	1.70 (1.44–2.01)	(6 studies)		
			(11.1 to 15.5)				
					
	Exclusively breastfeeding infants, who ever initiated breastfeeding, surviving 2–4 days	<24 Hours:	≥24 Hours:		65,215	Moderate^5^	
		6.9 per 1000	12.4 per 1000	1.85 (1.29–2.67)	(4 studies)		
			(8.9 to 18.4)				
					
	Low birthweight infants, who ever initiated breastfeeding, surviving 2–4 days	<24 Hours:	≥24 Hours:	1.73 (1.38–2.18)	21,258	Moderate^5^	

^1^ The assumed risk is the median risk in the 'early breastfeeding' group across all studies providing this information.

^2^ The corresponding risk is based on the assumed risk in the 'early breastfeeding' group and the relative effect of the intervention (and its 95% confidence interval).

^3^ GRADE Working Group grades of evidence description [17]: High quality: Further research is very unlikely to change our confidence in the estimate of effect.; Moderate quality: Further research is likely to have an important impact on our confidence in the estimate of effect and may change the estimate.; Low quality: Further research is very likely to have an important impact on our confidence in the estimate of effect and is likely to change the estimate.; Very low quality: We are very uncertain about the estimate.

^4^ All five studies are categorized as having a moderate risk of bias, but the overall strength of evidence is upgrade to 'High' because the studies are consistent, there is a large effect size (RR >2), and there is evidence dose response.

^5^ All studies are categorized as having a moderate risk of bias. There is no evidence of dose response (due to study design) and there is no large effect size

All six studies of timing of breastfeeding initiation and nutritional status (e.g. stunting, wasting, underweight, or early weight loss) were considered very low quality, and the findings were inconsistent across the studies (S3 Table). We could not pool any of the estimates due to variations in the exposure definition or the time period of outcome assessment (S3 Table).

There were five studies which examined the relationship between timing of breastfeeding initiation on morbidity (*e*.*g*. diarrhea, respiratory infections, hypothermia, and umbilical cord infection) with mixed quality levels (Table 4). We were unable to pool any of the morbidity studies due to differences in exposure definition, time of outcome assessment, or difference in type of effect estimate (Table 4). The available information regarding diarrhea and respiratory infections was of low or very low quality. Mullany and colleagues provide moderate quality evidence regarding an association between delayed breastfeeding and an increased risk of umbilical cord infection among infants in Tanzania [31] and an increased risk of hypothermia among infants in Nepal [32]. Similarly, in a high quality study Van den Bosch and Bullough reported a two-fold greater risk of hypothermia among infants randomized to “mother’s choice of breastfeeding initiation time” compared to those randomized to immediate breastfeeding initiation in Malawi [35].

**Table 4:** Summary of studies of the association between early breastfeeding initiation and morbidity outcomes (*Reference group).

**Diarrhea**	** **	** **	** **	** **	** **	** **
Study	Sample Size	Study Design	Exposure Definition	Outcome Definition	Effect Estimate	Quality
Clemmens	198	Prospective	Early (<3 days) vs. Late (≥3 days)*	Diarrhea	aRR: 0.74 (95% CI: 0.56–0.98)	Low
1999		Cohort	breastfeeding initiation	at <6 months		
Clemmens	198	Prospective	Early (<3 days) vs. Late (≥3 days)*	Diarrhea	aRR: 0.95 (95% CI: 0.70–1.31)	Low
1999		Cohort	breastfeeding initiation	at 6–12 months		
Hajeebhoy	6068	Cross-Sectional	Early (<1 hr) vs. Late (>1 hr)*	Diarrhea	aOR: 0.74 (95% CI:0.58–0.93)	Very low
2014			breastfeeding initiation	at <6 months		
**ARI**	** **	** **	** **	** **	** **	** **
Hajeebhoy	6068	Cross-Sectional	Early (<1 hr) vs. Late (>1 hr)*	ARI	aRR: 0.91 (95% CI: 0.80–1.03)	Very low
2014			breastfeeding initiation	at <6 months		
**Hypothermia**	** **	** **	** **	** **	** **	** **
Mullany	19180	Prospective	Early (<24 hrs)* vs. Late (>24 hrs)	Prevalence of axillary measures <35.0°C at <28 days	aRR: 1.19 (95% CI: 1.08–1.30)	Moderate
2010		Cohort	breastfeeding initiation			
					
Van den Bosch	160	Randomized Trial	Immediate* vs. Mother's choice of breastfeeding initiation time	Rectal temperatue <36.5°C at 2, 4, and ~24 hrs after birth	RR: 2.45 (95% CI: 1.36–4.41)	High
1990						
				
**Umbilical Cord Infection**		** **	** **	** **	** **	** **
Mullany	1653	Prospective Cohort	Early (<1 hr) vs. Late (≥1 hr)*	1. Pus with any redness (Broad)	1. aRR: 0.74 (95% CI: 0.38–1.47)	Moderate

aRR

## Discussion

Our review provides new insight on the increased risk of neonatal mortality associated with delayed breastfeeding initiation (defined in this review as initiation after the first hour after birth). We demonstrated a clear dose-response relationship; the risk of neonatal mortality increased with increased delay in breastfeeding initiation. Infants who initiated breastfeeding between 2–23 hours after birth had a 33% greater risk of neonatal mortality compared to infants who initiated breastfeeding within an hour of birth. Neonatal mortality risk was more than 100% greater in infants who initiated breastfeeding more than 24 hours after birth. Our findings are based on five prospective cohort studies of 136,047 breastfed, live born infants who survived the first two to four days of life.

The intervention of interest (*i*.*e*. early breastfeeding initiation) has been inconsistently defined across studies. Some authors define “early breastfeeding initiation” as breastfeeding within one hour of birth (as we do here); others define early initiation as “within three days of birth” [23]. Two previously published meta-analyses defined early breastfeeding initiation as initiation within 24 hours of birth [4, 5]. However, in our three largest cohorts, accounting for nearly 100,000 infants [12–15], very few mothers initiated breastfeeding after 24 hours (n = 4,577). In addition, the World Health Organization (WHO) and the United Nations Children’s Fund (UNICEF) recommend that breastfeeding is initiated within an hour of birth. Thus, we proposed that the primary intervention of interest for this study should be breastfeeding initiation within an hour of birth (*i*.*e*. very early breastfeeding initiation). Using this definition of “very early breastfeeding initiation”, we pooled effect estimates for all-cause neonatal mortality for more than 136,000 infants enrolled in prospective cohorts in Ghana, India, Nepal, and Tanzania [12–15, 27, 33]. Similar relationships were demonstrated after pooling the six studies [12–15, 27, 30, 33] which assessed breastfeeding initiation <24 hours. We found that early initiation of breastfeeding was associated with a reduced risk of mortality, with a very similar effect size, even when the analysis was restricted to low birth weight infants. Due to the higher baseline risk of death among low birthweight infants, large gains in the number of deaths averted may be achieved through very early breastfeeding initiation in this group.

We also found that early initiation of breastfeeding was similarly associated with reduced risk of mortality, even when the analysis was restricted to exclusively breastfed infants. We have previously reported a similar finding regarding the relationship between breastfeeding initiation within an hour of birth and neonatal mortality in exclusively breastfed infants using pooled data from nearly 100,000 infants enrolled in neonatal vitamin A supplementation trials [15]. This pooled analysis demonstrated a strong association between very early breastfeeding initiation (within one hour of life) and reduced risk of mortality among infants who were exclusively breastfed in the neonatal period, at one month, and at three months of life [15]. This systematic review and meta-analysis found there was no additional data to pool beyond that provided by the neonatal vitamin A supplementation trials, as no other studies examined the effect of very early breastfeeding (within one hour) in exclusively breastfed infants. However, we updated the meta-analysis regarding early initiation of breastfeeding (within 24 hours) among exclusively breastfed infants, though we were unable to include the largest cohort (Mazunder 2015) as the number of exclusively breastfed infants who initiated breastfeeding more than 24 hours after birth was very small. The pooled results of four studies (including 65,215 infants) showed that initiation of breastfeeding after the first 24 hours of life was associated with an 85% increased risk of neonatal mortality compared to infants who initiated breastfeeding within 24 hours after birth, and there was no evidence of heterogeneity of effect. We previously postulated that early initiation of breastfeeding may independently reduce neonatal and early infant mortality by specific biological mechanisms, in addition to increasing rates of exclusive breastfeeding [15]. Our new meta-analysis provides additional evidence to support this hypothesis. This is in contrast to the previous meta-analysis which reported that there was no association between early breastfeeding (within 24 hours) and all-cause neonatal mortality among those that were exclusively breastfed [4]. However, as noted by Debes *et al*, there was limited data available to examine the exclusively breastfed subgroup at that time.

We identified five studies which examined the association between early breastfeeding initiation and morbidity (*e*.*g*. diarrhea, respiratory infections, hypothermia, and umbilical cord infection) [7, 23, 31, 32, 35], and there were six studies that examined nutrition outcomes [8, 11, 22, 24, 28, 29]. However, most papers had a ‘low’ or ‘very low’ quality score, and we were unable to pool the study-specific estimates due to differences in the exposure definition, time of outcome assessment, or type of published effect estimate. Additional, higher quality research is needed to understand the relationship between early breastfeeding initiation and infant morbidity and nutrition outcomes.

Our findings have important implications for prioritizing interventions to improve neonatal survival. There is a strong biological basis for potential mechanisms that might explain the survival benefits associated with early breastfeeding. Early breastfeeding initiation exposes the infant to maternal colostrum, which is thought to decrease the risk of microbial translocation, accelerate intestinal maturation, and promote resistance and epithelial recovery from infection [36, 37]. Early breastfeeding may also reduce hypothermia and foster attachment and bonding through close contact with the mother. Similarly, kangaroo mother care—an intervention including skin to skin contact, exclusive breastfeeding, early discharge from the hospital, and follow up care for infant[38]—has been shown to reduce the risk of hypothermia by 72% and reduce the risk of early mortality by 33% [39]. Since early breastfeeding inherently includes skin to skin contact between the newborn and mother, this may be one mechanism through which it could improve neonatal survival. Based on our review of the evidence, we recommend that early initiation of breastfeeding should be considered when estimating the overall survival benefits of breastfeeding, and should be considered for inclusion in models that assess the benefits of interventions for infant survival, such as those used in the Lives Saved Tool (LiST) [18].

There are several strengths of this systematic review and meta-analysis. First, we conducted a thorough review of the literature using all appropriate search engines, without limitations based on date of publication. For all outcomes, we provided a narrative synthesis of the evidence to account for the heterogeneity of exposure definition. Finally, the meta-analysis of the effect estimate for the relationship between breastfeeding initiation and all-cause infant mortality is based on large cohorts nested within well-conducted, population-based, randomized control trials, and we analysed the data to demonstrate the strength of a dose-response relationship. Our review and meta-analysis also had several limitations. We were unable to perform subgroup analyses by quality score, income status of country, or maternal HIV status as there were insufficient studies in these subgroups. Three studies [12–14], which have previously published pooled estimates [15], presented effect estimates for infant mortality between one to three months and three to six months, and no studies presented effect estimates for infant mortality through 12 months. The review was also based entirely on observational data. It is important to note that there are many reasons for delayed breastfeeding initiation that may confound the relationship between breastfeeding initiation and mortality. However, a randomized trial of this intervention would not be considered ethical, so we must rely on methodologically robust analysis of high quality observational data. Further, we did not specifically consider gestational age in this analysis. However, adjustment for gestational age or low birthweight was a required criteria for a study achieving moderate or high quality ranking. Most study estimates were based on models that included low birthweight, which is a good proxy for preterm birth in settings where gestational age dating by ultrasound is uncommon and gestational age dating by maternal recall of last menstrual period is unreliable. Additional research among high quality, prospective cohorts regarding the relationship between early breastfeeding initiation and cause-specific mortality and severe morbidity would strengthen the overall quality of the evidence.

Our study suggests that early breastfeeding initiation should be taken into account when policy frameworks or models such as *LiST* are applied to estimate the survival benefits of breastfeeding. Breastfeeding promotion programs which can remove structural, cultural, and information barriers to promote breastfeeding should also emphasize the importance of early initiation of breastfeeding, in addition to promoting exclusive breastfeeding. Furthermore, health facility policies and health provider knowledge can promote early breastfeeding initiation. This is particularly relevant for countries, where neonatal and infant mortality rates are high, most women already exclusively or predominantly breastfeed their infants, and delayed initiation of breastfeeding beyond the first hour of life is common.

## Supporting information

S1 Text Specific search strategy. (PDF)

S2 Text PRISMA checklist. (PDF)

S1 Table Summary of relevant studies. (PDF)

S2 Table Quality assessment of included studies. (PDF)

S3 Table Summary of studies of the association between early breastfeeding initiation and nutrition outcomes. (PDF)

S1 Fig Forest Plot of the relative risk of neonatal mortality (excluding deaths in the first 2–4 days) for infants who initiated breastfeeding 2–23 hours or ≥24 hours after birth, compared to those who initiated breastfeeding early (<1 or ≤1 hour)–including Garcia 2001 estimates. (PDF)
