# Supplementary material for: Delayed breastfeeding initiation and infant survival: A systematic review and meta-analysis
Source: PLoS One. 2017 Jul 26;12(7):e0180722. doi: 10.1371/journal.pone.0180722 (PMC5528898; doi:10.1371/journal.pone.0180722)
Supplement: S1 Text — (PDF) [file pone.0180722.s001.pdf]

S1 Text
Specific Search Strategy

Database: PubMed
Date of Search: 2015-12-09
Results: 2112 Records
Search Strategy:
("Breast Feeding"[Mesh] OR breast fe*[tiab] OR breastfe*[tiab])
AND
("Time Factors"[mesh] OR initiat*[tiab] OR timing[tiab] OR delay*[tiab] OR start[tiab] OR starting[tiab] OR starts[tiab] OR started[tiab] OR early breast* OR early suckl*[tiab])
AND
("Infant Mortality"[Mesh] OR "Infant Death"[Mesh] OR "Perinatal Death"[Mesh] OR "mortality"[Subheading] OR "Survival"[Mesh] OR mortality[tiab] OR death*[tiab] OR died[tiab] OR survival[tiab] OR "Diarrhea"[Mesh] OR diarrhea*[tiab] OR diarrhoea*[tiab] OR "Respiratory Tract Infections"[Mesh] OR respiratory infect*[tiab] OR respiratory illness*[tiab] OR respiratory disease*[tiab] OR respiratory tract infect*[tiab] OR influenza[tiab] OR pneumonia[tiab] OR bronchitis[tiab] OR "Sepsis"[Mesh] OR sepsis[tiab] OR septic[tiab] OR bacteremia[tiab] OR septicemi*[tiab] OR ("Umbilical Cord"[mesh] AND "Bacterial Infections and Mycoses"[Mesh]) OR omphalitis[tiab] OR umbilical cord infection*[tiab] OR "Hospitalization"[Mesh] OR hospitali*[tiab] OR hospital admission*[tiab] OR "Malnutrition"[Mesh] OR "Infant Nutrition Disorders"[Mesh] OR "Child Nutrition Disorders"[Mesh] OR "Wasting Syndrome"[Mesh:noexp] OR "Weight Gain"[mesh] OR "Weight Loss"[mesh] OR malnutri*[tiab] OR malnourish*[tiab] OR mal nutri*[tiab] OR under nourish*[tiab] OR undernutrition[tiab] OR under nutrition[tiab] OR nutritional status[tiab] OR weight gain[tiab] OR weight loss[tiab] OR underweight[tiab] OR under weight[tiab] OR growth failure[tiab] OR marasmus[tiab] OR kwashiorkor[tiab] OR stunt*[tiab] OR wasting[tiab] OR weight for age[tiab] OR length for age[tiab] OR weight for length[tiab])
AND
("Infant"[Mesh] OR "Child, Preschool"[Mesh] OR infant*[tiab] OR new born*[tiab] OR newborn*[tiab] OR neonat*[tiab] OR baby[tiab] OR babies[tiab] OR child*[tiab])

Database: Embase
Date of Search: 2015-12-09
Results: 684 Records
Search Strategy:
(('breast feeding'/exp AND 'time'/exp) OR (('breast fed' OR 'breast feed' OR 'breast feeds' OR 'breast feeding' OR breastfe* OR suckl*) NEAR/3 (initiat* OR timing OR delay* OR start OR starting OR starts OR started OR early OR immediate OR beginning)):ab,ti)
AND
('infant mortality'/exp OR 'newborn death'/exp OR 'perinatal death'/exp OR 'perinatal mortality'/exp OR 'survival'/exp OR mortality:ab,ti OR death*:ab,ti OR died:ab,ti OR survival:ab,ti OR 'infantile diarrhea'/exp OR diarrhea*:ab,ti OR diarrhoea*:ab,ti OR 'respiratory tract infection'/exp OR (respiratory NEAR/3 (infect* OR illness* OR disease*)):ab,ti OR influenza:ab,ti OR pneumonia:ab,ti OR bronchitis:ab,ti OR 'newborn sepsis'/exp OR sepsis:ab,ti OR septic:ab,ti OR bacteremia:ab,ti OR septicemi*:ab,ti OR 'omphalitis'/exp OR omphalitis:ab,ti OR (('umbilical cord' OR umbilicus) NEAR/3 infection*):ab,ti OR 'child hospitalization'/exp OR hospitali*:ab,ti OR (hospital NEAR/3 admission*):ab,ti OR 'malnutrition'/exp OR 'nutritional deficiency'/exp OR 'wasting syndrome'/exp OR 'weight gain'/exp OR 'weight loss'/exp OR malnutri*:ab,ti OR malnourish*:ab,ti OR undernourish*:ab,ti OR (mal NEXT/1 nutri*):ab,ti OR (under NEXT/1 nourish*):ab,ti OR undernutrition:ab,ti OR 'under nutrition':ab,ti OR 'nutritional status':ab,ti OR 'weight gain':ab,ti OR 'weight loss':ab,ti OR

underweight:ab,ti OR 'under weight':ab,ti OR 'growth failure':ab,ti OR marasmus:ab,ti OR kwashiorkor:ab,ti OR stunt*:ab,ti OR wasting:ab,ti OR 'weight for age':ab,ti OR 'length for age':ab,ti OR 'weight for length':ab,ti)
AND
('infant'/exp OR 'preschool child'/exp OR infant*:ab,ti OR (new NEXT/1 born*):ab,ti OR newborn*:ab,ti OR neonat*:ab,ti OR baby:ab,ti OR babies:ab,ti OR child*:ab,ti)

Database: Web of Science
Science Citation Index Expanded (SCI-EXPANDED) --1900-present
Social Sciences Citation Index (SSCI) --1900-present
Conference Proceedings Citation Index- Science (CPCI-S) --1990-present
Conference Proceedings Citation Index- Social Science & Humanities (CPCI-SSH) --1990-present
Date of Search: 2015-12-10
Results: 696 Records
Search Strategy:
TS=(("breast fed" OR "breast feed" OR "breast feeds" OR "breast feeding" OR "breastfe*" OR "suckl*") NEAR/3 ("initiat*" OR "timing" OR "delay*" OR "start" OR "starting" OR "starts" OR "started" OR "early" OR "immediate" OR "beginning"))
AND
TS=("mortality" OR "death*" OR "died" OR "survival" OR "diarrhea*" OR "diarrhoea*" OR ("respiratory" NEAR/3 ("infect*" OR "illness*" OR "disease*")) OR "influenza" OR "pneumonia" OR "bronchitis" OR "sepsis" OR "septic" OR "bacteremia" OR "septicemi*" OR "omphalitis" OR (("umbilical cord" OR "umbilicus") NEAR/3 infection*) OR "hospitali*" OR ("hospital" NEAR/3 "admission*") OR "malnutri*" OR "undernourish*" OR "mal nutri*" OR "malnourish*" OR "under nourish*" OR "undernutrition" OR "under nutrition" OR "nutritional status" OR "weight gain" OR "weight loss" OR "underweight" OR "under weight" OR "growth failure" OR "marasmus" OR "kwashiorkor" OR "stunt*" OR "wasting" OR "weight for age" OR "length for age" OR "weight for length")
AND
TS=("infant*" OR "new born*" OR "newborn*" OR "neonat*" OR "baby" OR "babies" OR "child*")

Database: CINAHL
Date of Search: 2015-12-10
Results: 327 Records
Search Strategy:
(MH ("Time Factors" AND MH "Breast Feeding+") OR TI (("breast fed" OR "breast feed" OR "breast feeds" OR "breast feeding" OR breastfe* OR suckl*) N3 (initiat* OR timing OR delay* OR start OR starting OR starts OR started OR early OR immediate OR beginning)) OR AB (("breast fed" OR "breast feed" OR "breast feeds" OR "breast feeding" OR breastfe* OR suckl*) N3 (initiat* OR timing OR delay* OR start OR starting OR starts OR started OR early OR immediate OR beginning)))
AND
(MH ("Infant Mortality" OR "Infant Death" OR "Perinatal Death" OR "Diarrhea") OR "Diarrhea (NANDA)" OR "Respiratory Tract Infections+" OR "Sepsis+" OR "Hospitalization" OR "Malnutrition" OR "Infant Nutrition Disorders" OR "Child Nutrition Disorders" OR "Nutitional Status" OR "Nutritional Status (Iowa NOC)" OR "Wasting Syndrome" OR "Weight Gain" OR "Weight Loss") OR TI ("mortality" OR "death*" OR "died" OR "survival" OR "diarrhea*" OR "diarrhoea*" OR ("respiratory" N3 ("infect*" OR "illness*" OR "disease*")) OR "influenza" OR "pneumonia" OR "bronchitis" OR "sepsis" OR "septic" OR "bacteremia" OR "septicemi*" OR "omphalitis" OR (("umbilical cord" OR "umbilicus") N3 infection*) OR "hospitali*" OR ("hospital" N3 "admission*") OR "malnutri*" OR "malnourish*" OR "undernourish*" OR "mal nutri*" OR "under nourish*" OR "undernutrition" OR "under nutrition" OR "nutritional status" OR "weight gain" OR "weight loss" OR "underweight" OR "under weight" OR

"growth failure" OR "marasmus" OR "kwashiorkor" OR "stunt*" OR "wasting" OR "weight for age" OR "length for age" OR "weight for length") OR AB ("mortality" OR "death*" OR "died" OR "survival" OR "diarrhea*" OR "diarrhoea*" OR ("respiratory" N3 ("infect*" OR "illness*")) OR "influenza" OR "pneumonia" OR "bronchitis" OR "sepsis" OR "septic" OR "bacteremia" OR "septicemi*" OR "omphalitis" OR (("umbilical cord" OR "umbilicus") N3 infection*) OR "hospitali*" OR ("hospital" N3 "admission*") OR "malnutri*" OR "malnourish*" OR "undernourish*" OR "mal nutri*" OR "under nourish*" OR "undernutrition" OR "under nutrition" OR "nutritional status" OR "weight gain" OR "weight loss" OR "underweight" OR "under weight" OR "growth failure" OR "marasmus" OR "kwashiorkor" OR "stunt*" OR "wasting" OR "weight for age" OR "length for age" OR "weight for length"))
AND
((MH "Infant+" OR "Child, Preschool") OR TI ("infant*" OR "new born*" OR "newborn*" OR "neonat*" OR "baby" OR "babies" OR "child*") OR AB ("infant*" OR "new born*" OR "newborn*" OR "neonat*" OR "baby" OR "babies" OR "child*"))

Database: POPLINE
Date of Search: 2015-12-17
Results: 744 Records
Note: Acknowledgements for assistance with running this search to Debra L. Dickson | POPLINE Manager | Knowledge for Health (K4Health) Johns Hopkins Center for Communication Programs (CCP), 111 Market Place, Suite 310, Baltimore, MD 21202 ddickson@jhuccp.org | www.popline.org | 410-659-6300
Search Strategy:

("breast feed" OR "breast feeds" OR "breast feeding" OR "breast fed" OR breastfe*)
AND
(initiation OR initiate* OR initiating OR timing OR delay*)
AND
(mortality OR death* OR died OR survival OR diarrhea* OR diarrhoea* OR "respiratory infection" OR "respiratory illness" OR "respiratory disease" OR "respiratory infections" OR "respiratory illnesses" OR "respiratory diseases" OR influenza OR pneumonia OR bronchitis OR sepsis OR septic OR bacteremia OR septicemi* OR omphalitis OR "umbilical cord infection" OR hospitali* OR "hospital admission" OR "hospital admissions" OR malnutri* OR malnourish* OR undernourish* OR undernutrition OR "nutritional status" OR "weight gain" OR "weight loss" OR underweight OR "under weight" OR "growth failure" OR marasmus OR kwashiorkor OR stunt* OR wasting OR "weight for age" OR "length for age" OR "weight for length")
AND
(infant* OR newborn* OR neonat* OR baby OR babies OR child*)

Database: LILACS
Date of Search: 2015-12-15
Results: 177 Records
Search Strategy:

((breast AND feed$) OR breastfe$ OR amamantamiento OR (aleitamento AND materno))
AND
(initiat$ OR timing OR delay$ OR inicia$ OR atrasar OR atraso OR retrasar OR retraso)
AND
(mortality OR death$ OR died OR survival OR diarrhea$ OR diarrhoea$ OR (respiratory AND (infection$ OR illnes$ OR disease&)) OR influenza OR pneumonia OR bronchitis OR sepsis OR septic OR bacteremia OR septicemi$ OR omphalitis OR hospitali$ OR (hospital AND admission$) OR

malnutri$ OR undernurish$ OR undernutrition OR marasmus OR kwashiorkor OR stunt$ OR wasting OR (weight AND (loss OR gain OR age OR length)) OR mortalida$ OR muerte OR supervivencia OR sobrevivencia OR diarrea OR ((infección$ OR enfermedad$) AND irespiratoria$) OR neumonía OR bronquitis OR septicemia OR onfalitis OR (ingres$ AND hospitalar$) OR desnutri$ OR ((aumento OR pérdida OR cambio OR bajo) AND peso) OR debilitante OR morte OR sobrevivência OR diarréia OR ((doença$ OR infecção) AND respiratóri$) OR gripe OR bronquite OR sepsia OR hospitalização OR ((Admiss$ OR internaç$) AND hospitalar$) OR subnutrição OR ((ganho OR perda OR mudança OR baixo OR abaixo OR idade OR comprimento) AND peso) OR nanismo OR desperdiçando)
AND
(infant$ OR newborn$ OR neonat$ OR baby OR babies OR child$ OR niño OR niños OR bebé OR bebés OR ((recién OR recém) AND nascido$) OR criança$)

Database: AIM
Date of Search: 2015-12-15
Results: 6 Records
Search Strategy:
((breast AND feed$) OR breastfe$) AND (initiat$ OR timing OR delay$) [keywords]

Database: Index Medicus for the Eastern Mediterranean Region - IMEMR
Date of Search: 2015-12-15
Results: 37 Records
Search Strategy:
((breast AND feed$) OR breastfe$) AND (initiat$ OR timing OR delay$) [KeyWords]
AND
(mortality OR death$ OR died OR survival OR diarrhea$ OR diarrhoea$ OR (respiratory AND (infection$ OR illnes$ OR disease&)) OR influenza OR pneumonia OR bronchitis OR sepsis OR septic OR bacteremia OR septicemi$ OR omphalitis OR hospitali$ OR (hospital AND admission$) OR malnutri$ OR undernurish$ OR undernutrition OR marasmus OR kwashiorkor OR stunt$ OR wasting OR (weight AND (loss OR gain OR age OR length))) [KeyWords]

Database: WPRI (Western Pacific)
Date of Search: 2015-12-15
Results: 37 Records
Search Strategy:
("breast feeding" OR breastfeeding) AND (initiation OR initiate OR initiates OR timing OR delay OR delays OR delayed)
