# Supplementary material for: Delayed breastfeeding initiation and infant survival: A systematic review and meta-analysis
Source: PLoS One. 2017 Jul 26;12(7):e0180722. doi: 10.1371/journal.pone.0180722 (PMC5528898; doi:10.1371/journal.pone.0180722)
Supplement: S1 Table — (PDF) [file pone.0180722.s003.pdf]

S1 Table. Summary of Relevant Studies

Study	Population	Study Design	Exposure Definition	Outcomes Definitions	Summary of Limitations	Quality Score
Akter 2015	Bangladesh Demographic and Health Survey	Cross-sectional	Early (<1 hr) vs. Late (>1 hr) breastfeeding initiation	Neonatal Mortality	Inadequate sample size (n=3190). Risk of selection bias due to cross-sectional study design. Risk of information bias due to long recall period (up to 3 years). Did not account for reverse causality.	Very low
Bamji 2008	India: Medak district, Andhra Pradesh	Retrospective (1998-2001) and prospective (2001-03) cohort	Early (<24 hrs) vs. Late (>=24 hrs) breastfeeding initiation	Neonatal Mortality	Risk of selection/attrition bias because 30% of selected women were not reachable for postpartum assessment. Risk of information bias due to long recall (1-2 months after birth). Inadequate adjustment for confounding. Did not account for reverse causality.	Very low
Caglar 2006	Turkey: Gaziosmanpasa Medical Faculty	Case control	Mean age at first breastfeeding (in hours)	Weight loss <10%	No adjustment for confounding.	Very low
Clemens 1999	Egypt : Abu Homos district	Prospective cohort study	Early (<3 days) vs. Late (>3 days) breastfeeding initiation	Diarrhea	Adjusted models do not include a term for preterm or low birthweight status.	Low
Dewey 2003	USA: Davis, California	Prospective cohort	Early (<1 hr) vs. Late (>1 hr) breastfeeding initiation	Excess weight loss at day 3	Inadequate adjustment for confounding. Did not account for reverse causality.	Very low
Edmond 2006	Ghana: Kintampo, Wenchi, Techiman, and Nkoranza districts in the Brong Ahafo region	Prospective cohort (within RCT)	Early (<1 hr) vs. Late (1-24 hr, 24-48 hr, 48-72 hr >=72 hr) breastfeeding iniation	Neonatal Mortality	None	Moderate
Engebretsen 2008	Uganda: Mbale district	Cross-sectional	Early (<2 hr) vs. Late (2-24 hr, >24 hr) breastfeeding iniation	Stunted (HAZ < –2 SD), Wasted (WHZ < –2 SD)	Risk of selection bias. Inadequate adjustment for confounding. Did not account for reverse causality.	Very low

S1 Table (continued). Summary of Relevant Studies

Study	Population	Study Design	Exposure Definition	Outcomes Definitions	Summary of Limitations	Quality Score
Enzunga 1990	Zaire: Evangelical Medical Centre	Prospective cohort	Time of breastfeeding initiation (hrs)	Weight loss since birth	The inclusion criteria and loss to follow up are not described. There is no information regarding assessment of exposure or outcome. There is no adjustment for confounding. Did not account for reverse causality.	Very low
Garcia 2011	India: Tamil Nadu	Cohort	Early (<12 hr) vs. Late (12-24 hrs, > 24 hrs) breastfeeding initiation	Neonatal Mortality	None	Moderate
Hajeebhoy 2014	Vietnam: 11 provinces	Cross-sectional	Early (<1 hr) vs. Late (>1 hr) breastfeeding initiation	Diarrhea<br>ARI	Risk of selection bias due to cross-sectional study design. Risk of information bias due to long recall period (up to 3.2 months). Inadequate adjustment for confounding. Did not account for reverse causality.	Very low
Meshram 2012	India: Medak district, Andhra Pradesh	Cross-sectional	Early (<1 hr) vs Late (1-3 hrs, 4-12 hrs, 13-24 hrs, >24 hrs) breastfeeding initiation	Stunted (HAZ < –2 SD), Underweight (WAZ<–2 SD),<br>Wasted (WHZ < –2 SD)	Inadequate sample size. Risk of selection bias due to cross-sectional study design. Inadequate adjustment for confounding.	Very low
Mullany 2008	Nepal: Sarlahi District	Prospective cohort (within a RCT)	Early (<1 hr) vs. Late (1-24 hr, 24-48 hr, 48-72 hr >=72 hr) breastfeeding iniation	Neonatal Mortality	None	Moderate
Mullany 2010	Nepal: Sarlahi District	Prospective cohort (within a RCT)	Breastfeeding initiation (hrs) <24 hrs and >=24 hrs	Hypothermia	None	Moderate
Mullany 2009	Tanzania: Pemba Island	Prospective cohort (within a RCT)	Early (<1 hr) vs. Late* (≥1 hr) breastfeeding initiation	Umbilical cord infection	None	Moderate
Neovita Study Group 2016 [Includes: Edmond 2015 Masanja 2015 Mazunder 2015]	Ghana, India, Tanzania: BrongAhafo Ghana, Haryana India, Dar es Salaam and Morogoro Tanzania	Prospective cohort (within 3 RCTs)	Early (≤1 hr) vs. Late (2-24 hr, >24 hr) breastfeeding iniation	Neonatal Mortality, Mortality from 1-3 months, Mortality from 3-6 months	None	Moderate

S1 Table (continued). Summary of Relevant Studies

Study	Population	Study Design	Exposure Definition	Outcomes Definitions	Summary of Limitations	Quality Score
Niswade 2011	India: Nagpur district	Cohort	Delayed initiation of breastfeeding (not defined)	Neonatal Mortality	Did not account for reverse causality.	Very low
Shah 2014	Bangladesh: Sylhet district	Prospective cohort (within RCT)	Early (<1 hr) vs. Late (>1 hr) breastfeeding initiation	Neonatal Mortality	None	Moderate
Sutan 2014	Indonesia: Aceh Province	Unmatched case control	Early (<1 hr) vs. Late (>1 hr) breastfeeding initiation	Neonatal Mortality	Risk of information bias due to restrospective assessment of breastfeeding after child death. Did not account for reverse causality.	Very low
Van den Bosch 1990	Malawi: Kamuzu Central Hsopital	Randomized Control Trial	Early initation encouragement vs. Initation at time of mother's choice	Hypothermia: Rectal temperatue <36.5°C at 2, 4, and ~24 hrs after birth	None. Note that randomized groups were comparable.	High
Wren 2015	Guatemala: Mam-Mayan communities in the Western Highlands	Cross-sectional	Early (<1 hr) vs. Late (>1 hr) breastfeeding initiation	Stunted (HAZ < –2 SD), Underweight (WAZ<–2 SD), Wasted (WHZ < –2 SD)	Inadequate sample size. Inadequate adjustment for confounding.	Very low
