# Supplementary material for: Delayed breastfeeding initiation and infant survival: A systematic review and meta-analysis
Source: PLoS One. 2017 Jul 26;12(7):e0180722. doi: 10.1371/journal.pone.0180722 (PMC5528898; doi:10.1371/journal.pone.0180722)
Supplement: S2 Table — (PDF) [file pone.0180722.s004.pdf]

S2 Table. Quality Assessment of Included Studies

Study	Score for study design	Risk of selection bias?	Risk of information bias?	Risk of attrition bias?	Adequate adjustment for confounding?	Accounted for reverse causality?	Should the score for study design be adjusted for study quality?	Score after adjustment
Akter 2015	Low - Cross-sectional	Yes - cross sectional	Yes - recall up to 3 years allowed	No - not applicable due to study design	Yes	No	Yes	Very low
Bamji 2008	Low - Retrospective and prospective cohort	Yes - 30% of selected women were not reachable and thus excluded from study	Yes - assessment conducted 1-2 months after delivery.	Yes - 30% of selected women were Not reachable and thus excluded from study	No - Proportions presented. No control for confounding.	Yes - excluded 7 children who died on day 1.	Yes	Very low
Caglar 2006	Low - Case control	No	No	No - not applicable due to study design	No	Yes	Yes	Very low
Clemens 1999	Low - Prospective cohort	No	No - median age at enrollment was 11 days (IQR 7-16).	No -loss to follow up = 10%	No - Adjusted models do not include preterm or low birthweight status.	Yes - Median age at enrollment was 11 days.	No	Low
Dewey 2003	Low - Prospective cohort	No	No	No	Not for our exposure and outcome of interest	Not for our exposure and outcome of interest	Yes	Very low
Edmond 2006	Low - Prospective cohort (within RCT)	No	No - median assessment at 14 days postpartum	No	Yes	Yes	Yes	Moderate
Engebretsen 2008	Low - Cross-sectional	Yes - cross sectional	No	No - not applicable due to study design	No	No	Yes	Very low
Enzunga 1990	Low - Prospective cohort	unclear	unclear	unclear	No	No	Yes	Very low

S2 Table (continued). Quality Assessment of Included Studies

Study	Score for study design	Risk of selection bias?	Risk of information bias?	Risk of attrition bias?	Adequate adjustment for confounding?	Accounted for reverse causality?	Should the score for study design be adjusted for study quality?	Score after adjustment
Garcia 2011	Low - Prospective cohort	No	No	No	Yes	Yes	Yes	Moderate
Hajeebhoy 2014	Low - Cross-sectional	Yes - cross sectional	Yes - median age as assessment was 3.3 months	No - not applicable due to study design	No	No	Yes	Very low
Meshram 2012	Low - Cross-sectional	Yes - cross sectional	No	No - not applicable due to study design	No	Yes	Yes	Very low
Mullany 2008	Low - Prospective cohort (within RCT)	No	No	No	Yes	Yes	Yes	Moderate
Mullany 2010	Low - Prospective cohort (within RCT)	No	No	No	Yes	Yes	Yes	Moderate
Mullany 2009	Low - Prospective cohort (within RCT)	No	No	No (89% of infants receive all 6 assessments)	Yes	Yes	Yes	Moderate
Neovita 2016 [Includes: Edmond 2015 Masanja 2015 Mazunder 2015]	Low - Prospective cohort (within RCT)	No	No	Very low LTFU	Yes	Early deaths excluded	Yes	Moderate
Niswade 2011	Low - Prospective cohort	No	No - visited on day 0 and day 1	No	Yes	No	Yes	Very low

S2 Table (continued). Quality Assessment of Included Studies

Study	Score for study design	Risk of selection bias?	Risk of information bias?	Risk of attrition bias?	Adequate adjustment for confounding?	Accounted for reverse causality?	Should the score for study design be adjusted for study quality?	Score after adjustment
Shah 2014	Low - Prospective cohort (within RCT)	No	No - visisted on day of birth	No (~10% exlcuded due to missing LMP)	Yes	Yes	Yes	Moderate
Sutan 2014	Low - Case control	No	Yes - retrospective assessment of breastfeeding after child death	No - not applicable due to study design	Yes	No	Yes	Very low
Van den Bosch 1990	High - Randomized Control Trial	No	No	No (~11% missing 24 hour measurement)	N/A (due to study design)	Yes - preterm, low birthweight, and low Apgar score infants excluded	Yes	High
Wren 2015	Low - Cross-sectional	Yes - cross sectional	No	No - not applicable due to study design	No	Yes	Yes	Very low
