# Supplementary material for: Delayed breastfeeding initiation and infant survival: A systematic review and meta-analysis
Source: PLoS One. 2017 Jul 26;12(7):e0180722. doi: 10.1371/journal.pone.0180722 (PMC5528898; doi:10.1371/journal.pone.0180722)
Supplement: S3 Table — (PDF) [file pone.0180722.s005.pdf]

S3 Table. Summary of studies of the association between early breastfeeding initiation and nutrition outcomes. (*Reference group)

Study	Sample Size	Study Design	Exposure Definition	Outcome Definition	Effect Estimate	Quality
**Malnutrition (Stunting / HAZ)**						
Wren 2015	190	Cross-Sectional	Early (<1 hr)* vs. Late (>1 hr) breastfeeding initiation	HAZ < −2 SD at <46 days	RR: 1.67 (95% CI: 1.15-2.43)	Very Low
Meshram 2012	351	Cross-Sectional	Early (<1 hr)* vs. Late (>1 hr) breastfeeding initiation	HAZ < −2 SD at 1 year	RR: 1.19 (95% CI: 0.63-2.26)	Very Low
Engebretsen 2008	723	Cross-Sectional	Early (<2 hrs)* vs. Late (2-24 hrs, >24 hrs) breastfeeding initiation	HAZ < −2 SD at <1 year	OR: (2-24 hrs): 1.06 (95% CI: 0.56-1.99)<br>OR: (>24 hrs): 1.27 (95% CI: 0.80-2.03)	Very Low
**Malnutrition (Wasting / WHZ)**						
Wren 2015	190	Cross-Sectional	Early (<1 hr)* vs. Late (>1 hr) breastfeeding initiation	WHZ < −2 SD at <46 days	RR: 0.84 (95% CI: 0.14-4.88)	Very Low
Meshram 2012	351	Cross-Sectional	Early (<1 hr)* vs. Late (>1 hr) breastfeeding initiation	WHZ < −2 SD at 1 year	RR: 0.85 (95% CI: 0.52-1.38)	Very Low
Engebretsen 2008	723	Cross-Sectional	Early (<2 hrs)* vs. Late (2-24 hrs, >24 hrs) breastfeeding initiation	WHZ < −2 SD at <1 year	OR: (2-24 hrs): 1.07 (95% CI: 0.39-2.92)<br>OR: (>24 hrs): 0.81 (95% CI: 0.33-2.06)	Very Low
**Malnutrition (Underweight / WAZ)**						
Wren 2015	190	Cross-Sectional	Early (<1 hr)* vs. Late (>1 hr) breastfeeding initiation	WAZ < −2 SD at <46 days	RR: 3.06 (95% CI: 1.49-6.29)	Very Low
Meshram 2012	351	Cross-Sectional	Early (<1 hr)* vs. Late (>1 hr) breastfeeding initiation	WAZ < −2 SD at 1 year	RR: 0.87 (95% CI: 0.59-1.28)	Very Low
**Malnutrition (Early weight loss)**						
Dewey 2003	280	Prospective Cohort	Early (<1 hr) vs. Late (>1 hr) breastfeeding initiation	Weight loss >10% since birth at 3 days	RR: 1.20 (95% CI: 0.62-2.33)	Very Low
Caglar 2006	90	Case-Control	Mean time to first breastfeeding (hrs)	Weight loss ≥10% since birth at 4-9 days	Weight loss ≥10% group: 3.89 hrs (SE 2.37)<br>Weight loss <10% group: 2.14 hrs (SE 1.31)	Very Low
Enzunga 1990	330	Prospective Cohort	Time of breastfeeding initiation (hrs)	Weight loss since birth	Time of breastfeeding initiation and weight loss were associated in univariate and multiavriate linear regression	Very Low
