# Supplementary material for: Delayed breastfeeding initiation and infant survival: A systematic review and meta-analysis
Source: PLoS One. 2017 Jul 26;12(7):e0180722. doi: 10.1371/journal.pone.0180722 (PMC5528898; doi:10.1371/journal.pone.0180722)
Supplement: S1 Fig — (PDF) [file pone.0180722.s006.pdf]

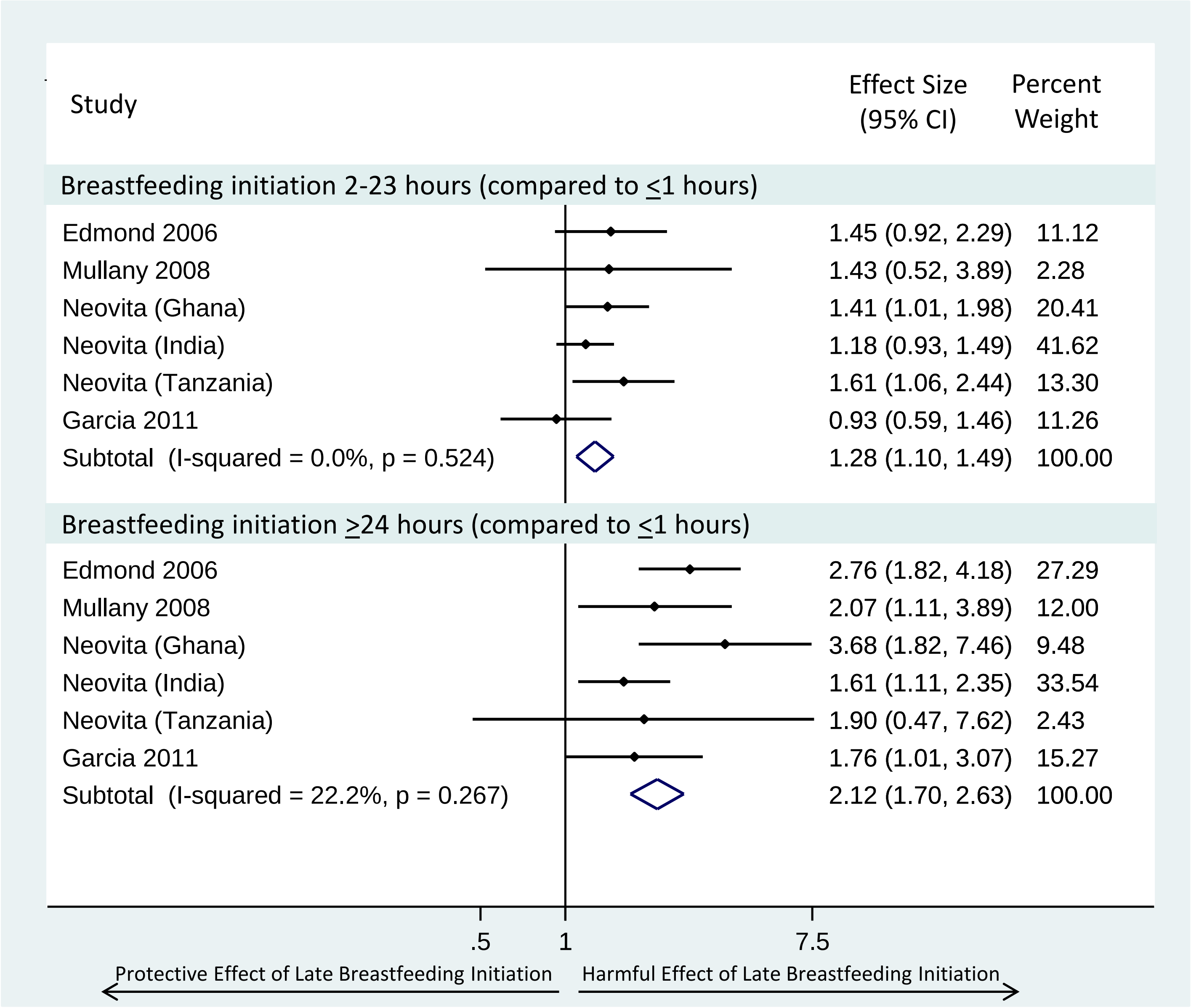

S1 Fig. Forest Plot of the relative risk of neonatal mortality (excluding deaths in the first 2-4 days) for infants who initiated breastfeeding 2-23 hours or >24 hours after birth, compared to those who initiated breastfeeding early (<1 or <1 hour) including Garcia 2001 estimates.
